# 

**Published:** 1890-09-06

**Authors:** 


SATURDAY, SEPTEMBER 6th, 1890.

**Free Trade in Medicine** - - - - - - - - 337

**Words of Consolation.**—Failure - - - - - - - 338

**The Doctor's Pulpit.**—More Life and Fuller - - - - 339

**At the Lords' Committee.**—By an Eye Witness - - - 340

**September** - - - - - - - - - - 341

**Working Women in Large Towns.** IX.—Domestic Servants 342

**Everybody's Page** - - - - - - - - 343

**Notes and News** - - - - - - - - 344

**The Attack on the Matron of the London.**—The Governors support the House Committee - - - - - 345

**Annotations.**—Kleptomania—The Cholera Scare—A New Tea 346

**The Practitioner's Mirror and Retrospect:—**

Medicine—Diarrhœa—Heart Disease in Children - - 347

Therapeutics—Antipyrin - - - - - - - - 348

A Medical Museum—Foods, Drugs, Instruments, &c. - - 348

The Practitioner's Bookshelf—Forensic Medicine - 349

New Drugs, Appliances, and Things Medical—Peter Möller's Cod Liver Oil—Mosely's "Compacta" Cabinets for Micro-Slides—"Bontha"—Bovinine - - 350

**Life in the New Hebrides** - - - - - - - - 351

**General Charities** - - - - - - - - - 352

**The Story of the Insane from Year to Year** - - 352

EXTRA SUPPLEMENT—THE NURSING MIRROR.

**En Passant.**—The Work of the Private Nurse—Louisa Alcott as Hospital Nurse—Catholic Nurses—The Melbourne Commission—The Nurse-Dolls—Our Duties to the Dying - - - - - - - - - xcv
**Sketches of Insanity.** By Francis H. Walmsley, M.D. IX.—Weak Mind - - - - - - - - - - xcvi
**Everybody's Opinion.**—Lady Nurses and Domestic Servants—A Nurse's Hours - - - - - - - - xcvii
**The Nurses' Bookshelf.**—A Novel for Nurses - - xcvii
**In Memoriam.**—Alice Fisher - - - - - - - xcviii
**Appointments—Notes and Queries—Vacancies** - xcviii
**Amusements and Relaxation** - - - - - - - xcviij

